# Molecular Characterization of Circulating Plasma Cells in Patients with Active Systemic Lupus Erythematosus

**DOI:** 10.1371/journal.pone.0044362

**Published:** 2012-09-21

**Authors:** Patricia L. Lugar, Cassandra Love, Amrie C. Grammer, Sandeep S. Dave, Peter E. Lipsky

**Affiliations:** 1 National Institutes of Health, Autoimmunity Branch, Bethesda, Maryland, United States of America; 2 Institute for Genome Sciences and Policy, Duke University, Durham, North Carolina, United States of America; Oklahoma Medical Research Foundation, United States of America

## Abstract

Systemic lupus erythematosus (SLE) is a generalized autoimmune disease characterized by abnormal B cell activation and the occurrence of increased frequencies of circulating plasma cells (PC). The molecular characteristics and nature of circulating PC and B cells in SLE have not been completely characterized. Microarray analysis of gene expression was used to characterize circulating PC in subjects with active SLE. Flow cytometry was used to sort PC and comparator B cell populations from active SLE blood, normal blood and normal tonsil. The gene expression profiles of the sorted B cell populations were then compared.

SLE PC exhibited a similar gene expression signature as tonsil PC. The differences in gene expression between SLE PC and normal tonsil PC and tonsil plasmablasts (PB) suggest a mature Ig secreting cell phenotype in the former population. Despite this, SLE PC differed in expression of about half the genes from previously published gene expression profiles of normal bone marrow PC, indicating that these cells had not achieved a fully mature status. Abnormal expression of several genes, including *CXCR4* and *S1P_1_*, suggests a mechanism for the persistence of SLE PC in the circulation. All SLE B cell populations revealed an interferon (IFN) gene signature previously only reported in unseparated SLE peripheral blood mononuclear cells. These data indicate that SLE PC are a unique population of Ig secreting cells with a gene expression profile indicative of a mature, but not fully differentiated phenotype.

## Introduction

SLE is a chronic autoimmune disease characterized by clinical and pathologic heterogeneity. It is well known that subjects with SLE have multiple B cell abnormalities and produce a variety of autoantibodies directed against nuclear, cytoplasmic and cell surface autoantigens [1], [2]. The B cell abnormalities include an increase in the number of circulating plasma cells (PC), disturbances in memory and naïve subsets, an activated phenotype and poor in vitro stimulation and survival [3]. The frequency of circulating PC correlates with disease activity, as measured by the SLEDAI scoring system, and the production of autoantibodies [4].

In the normal, non-diseased state, the frequency of circulating PC is quite low and increases in a tightly regulated manner following vaccination or infection [5]. However, in active SLE, the presence of PC is dysregulated with the persistent appearance of increased numbers in the circulation. The appearance of increased numbers of PC in the circulation of subjects with active SLE appears to reflect increased production in germinal centers, since circulating SLE PC contain highly mutated immunoglobulin (Ig) genes and their presence rapidly decreases following administration of a blocking monoclonal antibody to CD154 that limits T cell-B cell collaboration and germinal center formation [6]. The phenotypic markers of circulating SLE PC are CD27^++^CD38^++^CD19^lo^CD138^+^IgD^−^IgG^+^ [1], [6], [7].

Immunoglobulin secreting cells (IgSC) comprise a family of cells generated by a variety of in vivo stimuli and are indicative of different stages of maturation. Short-lived plasmablasts (PB) can derive rapidly from T-independent stimulation or as the initial IgSC induced during T cell-dependent responses. These cells secrete Ig for short periods of time before undergoing apoptosis, usually reside in the secondary lymphoid tissue in which they were generated and retain proliferative capacity. PB are thought to express CXCR3, which may allow them to migrate to inflammatory tissue [5]. Their occurrence in the circulation is very brief, however, and thus the study of circulating PB has been difficult [5]. Long-lived PC, in contrast, are terminally differentiated non-proliferative cells resulting from a T cell-dependent germinal center (GC) reaction in the follicles of lymphoid organs.

In GC, naïve B cells are stimulated by antigen in the presence of cognate T cell help to undergo proliferation accompanied by somatic hypermutation and Ig class switch recombination generating memory B cells or PC. Memory B cells retain a high affinity B cell receptor (BCR) at the cell surface but do not secrete antibody. They can respond rapidly with intense proliferation upon encountering antigen [8]. PC, however, have downregulated their BCR, and are non-dividing IgSC that do not require antigen encounter for survival or function [9]. After their generation in secondary lymphoid tissue, PC emigrate into the blood and then compartmentalize into tissue niches in the bone marrow and malt-associated lymphoid tissue where they persist and secrete Ig for long periods of time. Within these niches, PC continue to mature and differentiate into fully mature PC [10], [11].

These mature PC have a known pattern of gene expression, including the up-regulation of genes limiting apoptosis linked to the unfolded protein response and ER stress [12], [13]. Additionally, it is known that mature PC have upregulated expression of genes involved in cell cycle arrest and downregulated genes encoding specific B cell surface receptors, such as the BCR complex, B cell co-receptor (CD19, CD81, CD21), CD20 (*MS4A1*) and HLA Class II. Genes involved in PC homing to tissue niches such as CXCR4 and SDC1 are also up-regulated [14]. Increased expression of PRDM1 (encoding Blimp-1) is thought to be a master regulator of PC maturation by inducing or suppressing the expression of a number of genes involved in PC differentiation, such as *XBP1* and *BCL6*, respectively [14], [15], [16].

Although the exact regulatory process governing the B cell fate to differentiate into a memory B cell or an IgSC has not been completely delineated, a number of genes are known to be essential for PC differentiation and survival. Key regulatory transcription factors that initiate the differentiation to an IgSC are the expression of *PRDM1* (and encoding protein Blimp1), *XBP1*, *IRF4* and downregulation of earlier B cell lineage associated gene products by repression of regulatory transcription factors *SPIB*, *BCL6*, and *PAX5*. Moreover, down-regulation of a number of surface receptors, including MHC class II, BCR, CD19, CD21, CD22, CD40 and chemokine receptors (CXCR5, CCR7) and the upregulation of other surface receptors (CXCR4, SDC1) alter the function of PC.

We sought to utilize the known pattern of gene expression by human PC to investigate whether the circulating SLE PC shared a molecular program characterizing a prototypical PC and to gain additional insights into their maturational stage. We used tonsil PC and tonsil PB as comparator IgSC and normal peripheral blood naïve and memory B cells to compare similar B cell populations in patients with active SLE.

Here, we describe the results of the gene expression profiling of SLE PC in comparison to tonsil PC and PB, as well as naïve, pre-germinal center, germinal center and post germinal center tonsil B cells. Our data reveal that the prototypical signature that is known to characterize an IgSC is present in tonsil PC, tonsil PB and the SLE PC. Genes such *IRF4*, *PRDM1* and *XBP-1* are significantly upregulated and likewise the genes characteristic of the B cell or germinal center program such as *SPIB* and *BCL6* are significantly downregulated. In addition to the shared gene expression, we describe the unique characteristics of the SLE PC. This unique pattern of gene expression is found in SLE PC only and points to its aberrant persistence in the circulation. Additionally, the B cell lineage cells, including the PC of SLE share a type 1 interferon signature that is unique to SLE and not found in normal IgSC or circulating normal naïve and memory B cells.

## Methods

### Peripheral Blood and Tonsil Populations

Tonsil B cell populations were obtained from young patients (age 2–10) undergoing routine tonsillectomy with the use of a IRB protocol approved by the Clinical Center at the National Institutes of Health (Bethesda, MD) in accordance with the precepts established by the Declaration of Helsinki. The tonsils of these patients were disaggregated and separated by Ficoll gradient centrifugation. The mononuclear cell layer was harvested, washed in phosphate-buffered saline (PBS), and resuspended in ACK lysing buffer to remove small numbers of red blood cells. After washing and resuspension in 10 mL PBS with 10% bovine serum albumin, cells were counted and prepared for staining for flow cytometry as previously described [17], [18]. SLE patient samples were collected by leukapheresis of patients with active disease seen at the NIH clinical center. We used a SLEDAI score of greater that 4 to denote active disease. SLEDAI scores ranged between 4 and 10. Normal healthy adult donors, screened as part of the NIH healthy donor program, were subjected to leukapheresis as controls. All SLE and normal donor B cells were approved for research via a protocol approved by the Clinical Center at the National Institutes of Health (Bethesda, MD) in accordance with the precepts established by the Declaration of Helsinki. A total of at least three replicates for each B cell population (healthy tonsil, normal blood and SLE blood) are presented in this analysis.

### Flow Cytometric Separation and Analysis of B cell subpopulations

Peripheral blood mononuclear cells, PBMCs, were stained with mouse anti-human IgD FITC (Pharmingen) or goat anti-human IgD FITC (Caltag) and mouse anti-human CD19 PE (Becton Dickinson). Each of the SLE subjects had increased numbers of circulating plasma cells (PC) phenotypically expressing CD19^dim^IgD^−^CD38^++^.

The tonsillar mononuclear cells were incubated with mouse anti-human IgD FITC (Pharmingen) or goat anti-human IgD FITC (Caltag), anti-CD38 APC (BD Biosciences) and mouse anti-human CD19 PE (Becton Dickinson). Stained cells were analyzed with the use of the FACS Calibur (Becton Dickinson) or CyAN (DAKO-Cytomation, Fort Collins, CO) or sorted with a MoFlo Cell Sorter (DAKO). Paint-a-Gate (Becton Dickinson), CellQuest (Becton Dickinson) and Summit (DAKO-Cyomation) were used to analyze data generated by flow cytometry.

In SLE peripheral blood, PC were identified as CD19^dim^IgD^−^, whereas memory cells were CD19^+^IgD^−^ and naïve cells were CD19^+^IgD^+^. Further analysis of CD27 and CD38 expression demonstrated the uniformity of these populations. In the tonsil, PC were identified as CD19^+^CD38^+++^IgD^−^, plasmablasts as CD19^+^CD38^+++^IgD^+^, naïve cells as CD19^+^CD38^+^IgD^+^, memory cells as CD19^+^CD38^+/−^IgD^−^, dark zone cells/germinal center (GC) as CD19^+^CD38^++^IgD^−^ and pre-GC/germinal center founder cells as CD19^+^CD38^++^IgD^+^.

Sorted B cell populations with purity of greater than 99% were used for RNA extraction.

### Microarray analysis of gene expression

Sorted B cell subpopulations were placed in TRIZOL for RNA extraction (Invitrogen, Carlsbad, California) following the manufacturer's instructions. Isolated RNA was further purified with the RNeasy mini Kit (Qiagen, Valencia, California) and processed for microarray analysis using the standard Affymetrix protocols (www.affymetrix.com). Briefly, 1–10 µg RNA was reversed transcribed into cDNA (Invitrogen, Carlsbad, California). The template cDNA was purified for amplification and in vitro transcription to cRNA using BioArray™ HighYield™ RNA Transcript Labeling Kit (T7) (Enzo Life Sciences, Inc., Farmingdale, NY). cRNA was biotin labeled, purified and hybridized to HG-U133A Affymetrix Genechips®. Genechips® were scanned on a high resolution Affymetrix scanner using GCOS version 1.2 software.

Data analysis was conducted after standard Affymetrix algorithm analysis (MAS5).

### Data and Statistical analysis

The Ig secreting signatures of tonsil PC and PB were defined by comparing the tonsil PC to the tonsil naïve, pre-germinal center/germinal center founder (GCF), germinal center (GC) or dark zone, and post germinal center memory B cells to determine the differential gene expression. These populations are defined based on cell surface markers referred to in the methods. This data set was determined to be the PC signature of a prototypical Ig secreting cell and was used to compare the relative gene expression in SLE PC. The differential gene expression of SLE PC was determined after comparison with the naive and memory peripheral blood B cells in SLE and then compared gene by gene for relative expression in tonsil PC and tonsil PB. Similarly these populations are defined based on cell surface markers referred to in the methods.

The SLE naïve and memory B cells were compared with normal naïve and memory B cells to establish the unique signature observed in SLE.

A specific mRNA was considered to be differentially expressed after analysis using SAM, significance analysis of microarrays with 1000 permutations and a false discovery rate (q) less than 5% [19]. Differentially expressed mRNAs in tonsil PC, SLE PC and SLE naïve and memory B cells versus comparators were identified by the use of SAM. A two-tailed Students t-test was applied to individual comparisons only where stated when querying expression in specific gene sets. Bonferroni correction was applied to correct for multiple comparisons.

To visualize the individual gene expression levels within each sample, each gene is represented as a color with bright red representing the greatest expression level, bright green the least and black as equivocal. The genes that were significant in each comparison were tabulated and the median of the gene expression value was computed for each gene across the each column or sample in the heatmap. The calculation of the median allows the determination of the relative expression and color intensities as described previously [20].

Individual queried genes of interest and not differentially expressed genes where stated in text; were analyzed by converting the raw data to log2 values and then averaging across samples. The log2 transformed values were graphed as shown and p values derived using a student's t-test for statistical significance. When t-test was applied to determine significance in comparison of different B cell populations Bonferroni correction was applied to correct for multiple comparisons.

The stated gene function presented in the tables was gathered from Gene (NCBI) and HPRD (Human Protein Reference Database).

The data are accessible through Gene Expression Omnibus Series accession number GSE12845 at http://www.ncbi.nlm.nih.gov/geo/query/acc.cgi?acc_).

### Bone marrow plasma cell gene expression

Bone marrow PC and tonsil PC data for comparison were excerpted from published and publicly available data [21], [22]. The raw data was log2 transformed. Data with inconsistent probe sets or expression values below 6 after log2 transformation were not used for analysis and comparison with our data, according to our own standards of quality control for microarray data. The Genechips from published data were Affymetrix and similar to the Genechips used in the current study. Thus, probe sets were determined to be exact prior to comparisons after consultation with Affymetrix probe set conversion online at Affymetrix.com.

## Results

### Molecular Characterization of Ig Secreting Cells

**Figure 1:**
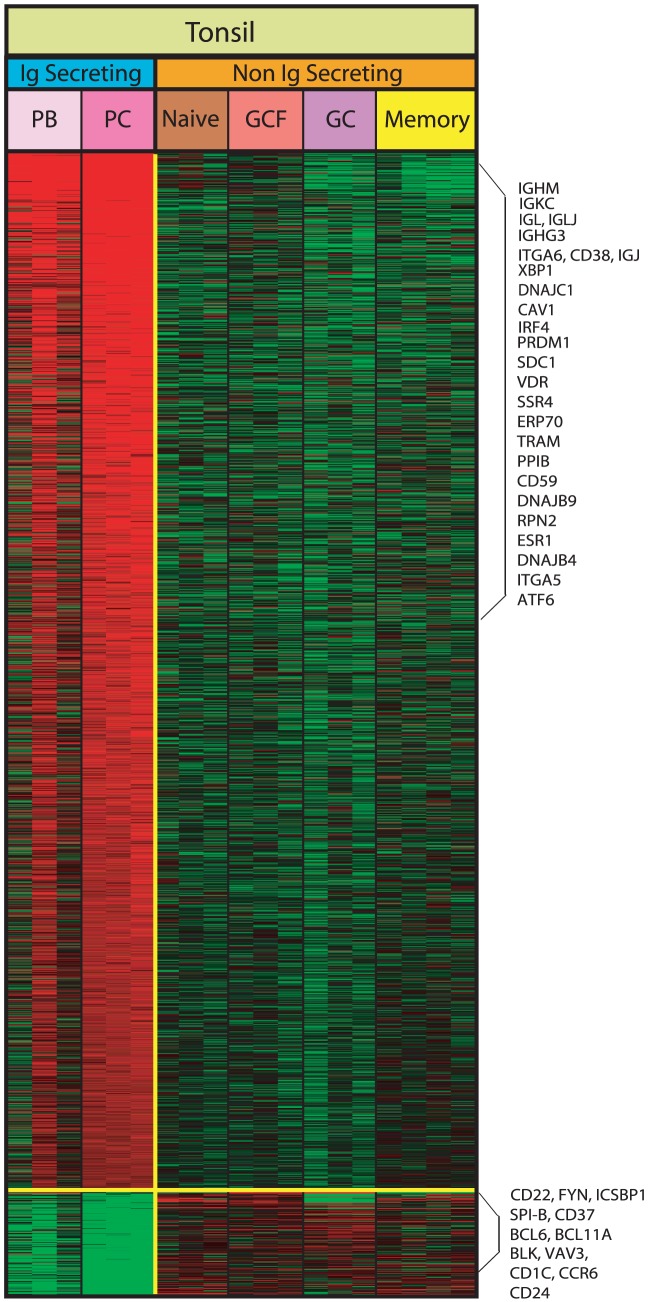
Gene expression profiling of sorted tonsil B cell populations. The heatmap shown represents the differential gene expression identified using- SAM in tonsil PC (TPC), tonsil naïve, pre-GC/germinal center founder (GCF), dark zone/germinal center (GC) and memory B cells. A total of 1858 genes are shown. 1690 are upregulated and 168 are downregulated. The majority of the gene signature is shared with tonsil PB (TPB).

To verify the molecular characteristics of tonsil PB and PC, we first performed gene expression profiling of tonsillar B cell populations. **Figure 1** demonstrates the genes upregulated and downregulated in tonsil PC (CD19^+^CD38^+++^IgD^−^) compared with the remaining tonsillar B cell populations (naïve cells CD19^+^CD38^+^IgD^+^, pre-GC/germinal center founder cells CD19^+^CD38^++^IgD^+^, dark zone cells CD19^+^CD38^++^IgD^−^ and memory cells CD19^+^CD38^+/−^IgD^−^). 1858 genes were differentially expressed in tonsil PC compared with non-Ig secreting tonsil B cells. Among the most prominent of the differentially expressed genes are transcription factors, repressors and other regulatory genes known to comprise the prototypical signature that characterizes an IgSC. Genes known to alter the B cell program and commit a B cell to PC differentiation, such *IRF4*, *PRDM1* and *XBP-1* were significantly upregulated and likewise the genes characteristic of the B cell or germinal center program such as *SPIB* and *BCL6* were significantly downregulated as well as *BLK*, *FYN*, *BCL11A*, *CD37*, *CD1C*, *ICSBP1*, *VAV3*, *CCR6* and *CD22*. Ig heavy and light chains as well as J chain were also significantly upregulated as well as *VDR*, *CAV1*, *SDC1*, *SSR4*, *ERP70*, *PPIB*, *ITGA6*, *CD38* and others denoted in **Figure 1** as previously reported to be PC genes [22], [23], [24], [25], [26], [27], [28]. The entire gene list in the order the genes appear in the heatmap of **Figure 1** are listed in **Table S1**.

Tonsil PB (CD19^+^CD38^+++^IgD^+^) were also isolated from the tonsil specimens. In **Figure 1**, the corresponding gene expression in tonsil PB of the 1858 differentially expressed tonsil PC genes is shown. Not surprisingly, tonsil PB gene expression pattern is highly similar to the tonsil PC. It is notable, however, that tonsil PB had more variability with respect to the degree of expression of the upregulated and downregulated genes distinguishing tonsil PC from non-IgSC of the tonsil. The differences in the gene expression between the tonsil PC and tonsil PB included greater relative expression of *ESR1*, *CAV1*, *SDC1*, *ITGA5*, *-6*, *ATF6*, *IRF4* and *XBP1* in tonsil PC, whereas tonsil PB had a greater relative expression of *CD22*, *CD24*, *ICSBP1*, *BLK*, *FYN* and *SPIB*, suggesting that they might be less mature. In order to demonstrate the relative expression of tonsil Ig secreting prototypical plasma cell genes, we examined a selected list of the commonly known most discriminating tonsil PC differentially expressed genes to compare expression in tonsil plasmablasts. **Table 1** shows the selected list of genes comprising the 1858 tonsil PC differentially expressed genes (both up- and downregulated compared with tonsil non-IgSC) and the relative gene expression in tonsil PB. The table highlights the differences in expression of these prototypical IgSC in tonsil. Considering the overall similarity of these two populations, we considered a fold change of 1.5 to determine upregulated and downregulated genes between tonsil plasma cells and plasmablasts. Fold change is included in the table to show striking differences in some key genes. The genes most notably upregulated in tonsil PC with respect to tonsil PB include chaperones, as well as cell cycle arrest and unfolded protein response (UPR) genes. The surface markers and transcription factors that characterize the mature B cell program are likewise underexpressed in all IgSC, but to a greater degree in tonsil PC than in tonsil PB, potentially also pointing to differences in maturational stage.

**Table 1:** TPC differentially expressed genes and comparative expression in TPB.

Genes Upregulated in Tonsil PC compared with Tonsil PB			Genes Equal in Expression in Tonsil PC and PB		
Gene Symbol	Fold change	Function	Gene symbol	Fold change	Function
**ATF6**	3.9	Unfolded Protein Response	*CCR6*	1.1	Chemokine receptor
**CASP10**	2.5	Apoptosis	*CD37*	0.8	Cell surface, complexes with integrins
**CAV1**	4	Intracellular signaling	**CD59**	0.9	Cell surface, complexes with integrins
**CD38**	2.5	Cell surface, intracellular signaling	**CD9**	1.2	Cell surface, intracellular signaling
**CDK5R2**	10.6	Cell cycle	**DNAJB9**	1.3	Chaperone
**CDKN1C**	3.6	Cell cycle arrest	**DNAJC3**	0.9	Chaperone
**CHST2**	1.9	Post translational modification	**FKBP11**	1.3	Chaperone
**CHST4**	3	Post translational modification	**GADD45A**	1.1	Cell cycle
**CHST8**	1.8	Post translational modification	**ITGB3**	1.3	Integrin
**DNAJB4**	1.7	Chaperone, Unfolded protein response	**MAN1A2**	1.2	Post translational modification
**DNAJB5**	1.5	Chaperone, Unfolded protein response	**PECAM1**	1.1	Chemotaxis and cellular migration
**DNAJB9**	1.5	Chaperone, Unfolded protein response	**PRDM1**	1.4	Transcription factor
**DNAJC1**	2.3	Chaperone, Unfolded protein response	**RPN1**	1.3	Post translational modification
**ESR1**	11	Transcription factor	**RPN2**	1.2	Post translational modification
**GADD45B**	1.8	Cell cycle arrest	**SSR1**	1.3	Protein assembly and trafficking
**GAS1**	4	Cell cycle arrest	**SSR4**	1.1	Protein assembly and trafficking
**GATA2**	1.9	Transcription factor	**TNFRSF17**	1.1	Inhibitor of apoptosis
**ICAM2**	2	Chemotaxis and cellular migration	*TNFSF11*	1.1	Inhibitor of apoptosis
**ID1**	1.7	Inhibitor of DNA transcription	**TRA1**	1	Chaperone
**IRF4**	1.9	Transcription factor	*TRAF5*	0.8	Signal transduction
**ITGA5**	1.9	Integrin	**TRAM1**	1.3	Protein assembly and trafficking
**ITGA6**	2.7	Integrin			
**IGF1R**	3.7	Inhibitor of apoptosis	**Genes Downregulated in Tonsil PC compared with Tonsil PB**		
**LTK**	3.4	Tyrosine kinase, intracellular signaling	**Gene symbol**	**Fold change**	**Function**
**XBP1**	1.6	Transcription factor	*BCL11A*	1.9	Transcriptional repressor
**SDC1**	2.1	Cell surface, intracellular signaling	*BLK*	4.4	Tyrosine Kinase
**SORT1**	2.3	Cellular signaling	*CD1C*	7.5	Cell surface
**MAN1A1**	2.6	Post translational modification	*CD22*	2.1	Cell surface, intracellular signaling
**TNFRSF4**	3	Inhibitor of apoptosis	*CD24*	6.8	Cell surface, intracellular signaling
**TRADD**	1.8	Inhibitor of apoptosis	**CD63**	1.5	Cell surface, complexes with integrins
**SSR3**	1.6	Protein assembly and trafficking	*ICSBP1*	1.8	Transcription factor
**HSPA13**	1.5	Chaperone, Unfolded protein response	**IGF1**	1.5	Signal transduction
**SVIL**	4.8	Chemotaxis and cellular migration	**CADM1**	1.6	Cell surface, intracellular signaling
			*IL7*	4.5	Cytokine
			*SPIB*	2.5	Transcription factor
			**TLR8**	2.4	Cell surface, intracellular signaling
			**TNFRSF11B**	2.2	Cell surface, intracellular signaling
			*FYN*	3.1	Tyrosine Kinase

Selected list of differentially expressed genes (using SAM) in tonsil plasma cells (CD19^+^CD38^+++^IgD^−^) compared to tonsil non-Ig secreting cells (naïve cells CD19^+^CD38^+^IgD^+^, memory cells CD19^+^CD38^+/−^IgD^−^, dark zone cells CD19^+^CD38^++^IgD^−^ and pre-GC/germinal center founder cells CD19^+^CD38^++^IgD^+^) and the relative expression in tonsil plasmablast (CD19^+^CD38^+++^IgD^+^). This list depicts the upregulated or downregulated genes in tonsil PC compared to tonsil PB. Bold typeface denotes a differentially upregulated gene in TPC compared with non IgSC from tonsil. Italics typeface denotes a differentially downregulated gene in TPC compared with non IgSC from tonsil. Gene function adapted from publicly available databases NCBI Gene and HPRD (Human Protein Reference Database).

### Molecular Characterization of the SLE PC

**Figure 2:**
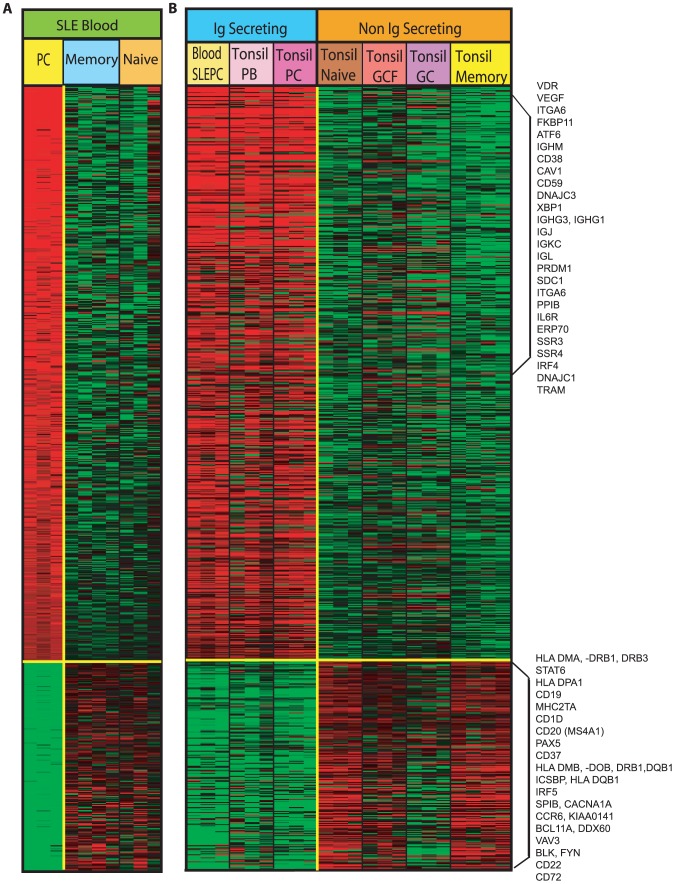
Gene expression profiling of circulating SLE B cells populations demonstrating the differential gene expression in SLE PC compared with SLE naïve and memory B cells. **A**. 780 genes are differentially expressed in SLE PC compared to SLE naive and memory B cells identified using SAM. 571 genes shown in the heatmap are upregulated and 209 genes are downregulated in SLE PC compared to SLE naïve and memory B cells. **B**. The genes differentially expressed in SLE PC were then compared gene by gene for corresponding expression in sorted tonsillar B cell populations.

Once the IgSC signature had been defined in tonsil, we next examined the gene expression profile of the circulating SLE PC. First, we searched for differentially expressed genes in the SLE PC compared with the circulating SLE naïve and memory B cells using SAM as described previously. **Figure 2a** demonstrates the 780 genes that are differentially upregulated and downregulated in SLE PC compared with SLE naïve and memory B cells. The genes in the upregulated signature included *XBP-1*, *IRF4*, *PRDM1*, *VDR*, *ERP70*, *CAV1*, *CD38*, *SDC1*, *SSR4*, *PPIB*, *ITGA6* and Ig heavy and light chains. The downregulated genes included *SPIB*, *BLK*, *FYN*, *ICSBP1*, *BCL11A*, *VAV3*, *CD1C*, *CD37*, *CCR6*, *BCL6* and *CD22*. These are the same differentially upregulated and downregulated genes seen in tonsil PC compared to the other tonsil B cell populations. *STAT6*, *IRF5*, HLA class II, *MHC2TA*, *TNFSF12*, *TNFSF13*, CD20 (*MSA4S1*), *CD72* and *CD19* were also differentially downregulated in SLE PC, and not seen as tonsil PC differentially expressed genes in **Figure 1**. The gene expression profile of the SLE PC demonstrates a pattern corresponding to the PC signature observed in the tonsil. The relative expression of 780 SLE PC genes with respect to tonsil PC, tonsil PB and the remaining tonsillar B cells is shown in **Figure 2b**, in which the SLE PC differentially expressed genes and the comparative gene expression in tonsillar B cell populations are shown. The differentially expressed SLE PC genes can be compared gene by gene to each B cell population of the tonsil (**Figure 2b**). As shown in the figure, the gene expression of SLE PC is quite similar to that of tonsil IgSC and is very different from the tonsil non-Ig secreting B cell populations. A complete list of the 780 differentially expressed SLE PC genes are provided in the order they appear in the heatmap of **Figure 2 A** and **B** in **Table S2**.

### Differences in the SLE PC from tonsil IgSC

**Figure 3:**
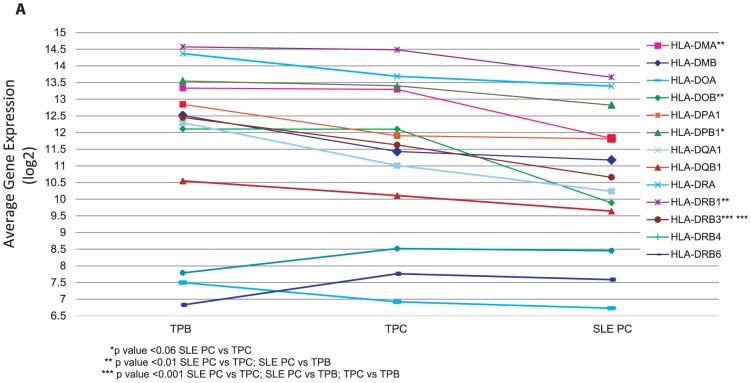
Unique gene expression pattern of HLA class II genes in SLE PC in comparison to tonsil PC and PB. Expression of HLA Class II genes in SLE PC, tonsil PC and tonsil PB. Differences in gene expression are shown as mean expression value log2 transformed data. Significance determined by student's t-test is denoted by asterisk(s) in the figure. Correction for multiple comparisons performed using Bonferroni correction.

We next sought to determine whether there were differences in gene expression unique to the circulating SLE PC and thus distinct from tonsil PC and tonsil PB that may play a role in their persistence in the circulating blood. Although the overall gene expression defining SLE PC is similar to tonsil IgSC with respect to key regulatory PC genes, the SLE PC also had a unique gene expression profile not seen in tonsil IgSC as shown in **Figure 2b**. One prominent difference in SLE PC is the downregulation of HLA class II genes. We, therefore, looked at all the HLA Class II genes on the HG-U133A Affymetrix Genechips® and the change in expression comparatively seen in SLE PC as compared with tonsil PC and tonsil PB. All HLA Class II genes were underexpressed in SLE PC as compared with tonsil PC and tonsil PB with the exception of *HLA –DRB4* and *HLA-DRB6* (**Figure 3**). The genes with a difference in expression that are statistically significant are shown in the figure and include the genes seen in **Figure 2b**.

We next characterized the degree of overlap in total gene expression between SLE PC and either tonsil PC or tonsil PB. **Figure 2b** demonstrates the degree of variability in the expression of genes in tonsil PC and tonsil PB from SLE PC, and indicates that tonsil PC and SLE PC may share an expression pattern more close to each other than to the tonsil PB.

**Figure 4:**
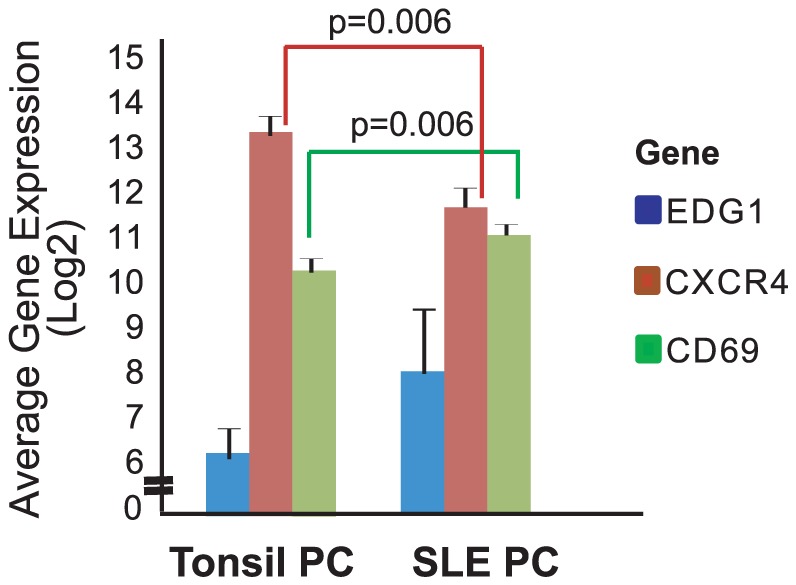
Relative expression of genes involved in lymphocyte trafficking. S1P_1_, CD69 and CXCR4 were examined for gene expression in IgSC populations (tonsil PC and SLE PC). A significant difference (p<0.06) in gene expression among the populations is denoted in the figure and was determined by a student's t-test with a correction for multiple comparisons using Bonferroni correction.

We next examined all of the genes differentially expressed in SLE PC compared to either tonsil PC or tonsil PB using SAM as previously described. **Table 2** lists the 77 genes that are differentially expressed in SLE PC compared to tonsil PC and the 72 genes differentially expressed in SLE PC compared with tonsil PB. The genes upregulated (shown in bold) in SLE PC compared with tonsil PC include genes involved in cell maintenance and proteins promoting survival and limiting apoptosis. A greater number of genes were downregulated in SLE PC (shown in italics) compared with tonsil PC and included signaling genes, genes involved in the promotion of apoptosis, cell growth and migration. It is notable that differentially expressed genes in SLE PC compared with tonsil PB are all downregulated in SLE PC. This list includes genes involved in cell metabolism, signaling, interactions with extracellular matrix, migration and induction of apoptosis. These differentially expressed tonsil plasmablasts genes are in concordance with their biology as circulating Ig secreting cells that home to and reside in inflamed or infected tissue, with a limited life span and prone to apoptosis. PC are persistently elevated in the blood of subjects with active SLE and thus we investigated expression of specific genes involved in PC trafficking and homing including *CXCR4*, *CD69* and *S1P_1_* in SLE PC and tonsil PC [29], [30], [31], [32]. **Figure 2** shows CXCR4 was downregulated as a differentially expressed SLE PC gene. We sought to determine if CD69 and S1P_1_ were similarly aberrantly expressed as possible contributing factors in abnormal SLE PC homing. **Figure 4** demonstrates the gene expression of *CXCR4*, *CD69* and *S1P_1_* in SLE PC and tonsil PC. *CXCR4* gene expression was significantly downregulated in SLE PC as compared with tonsil PC (p value 0.002). The significant downregulation of *CXCR4* was not only present in SLE PC compared with tonsil PC, but also when SLE naïve and memory B cells were compared with normal blood naïve and memory B cells (p value 0.005, 0.05 respectively- data not shown). *CD69* expression in SLE PC was significantly higher compared with tonsil PC (p value 0.002). This is unique to the PC compartment because other SLE B cell populations exhibited comparable expression to their normal counterparts (data not shown). This is interesting considering interferon alpha regulation of CD69 expression [32], [33] and reports of interferon alpha contributing to the pathogenesis of SLE. *S1P_1_* expression was also higher in SLE PC than in tonsil PC, but this was not significant.

**Table 2:** Differentially expressed genes in SLE PC compared to tonsil Ig secreting cells.

A	Differentially expressed genes in SLE PC compared to tonsil plasma cells			B	Differentially expressed genes in SLE PC compared to tonsil plasmablasts		
	Gene Symbol	Function	Fold change		Gene Symbol	Function	Fold Change
	**BACE2**	Protein metabolism	**10.6**	*	*ALDH2*	Metabolism	*16.0*
	**CDK2**	Cell cycle	**5.1**		*AP3D1*	Cellular trafficking	*9.1*
	**CENPN**	Cell cycle	**12.8**		*APOC1*	Cholesterol metabolism	*61.1*
	**CLIC3**	Ion transport	**12.8**		*APOE*	Cholesterol metabolism	*52.4*
	**CRIP1**	Ion transport	**21.9**	*	*ASB13*	Intracellular signaling	*10.7*
	**FGR**	Signal transduction	**19.1**		*CAMP*	Cathelicidin antimicrobial protein	*9.0*
	**GJA10**	Intercellular transport	**4.4**	*	*CCDC88A*	Cell growth, migration	*11.0*
	**HIST1H4K**	Transcription regulator	**19.4**		*CCL18*	Chemokine	*99.3*
	**PAK7**	Signal transduction	**9.9**	*	*CD9*	Signal transduction	*25.2*
	**RNPS1**	Transcription regulator activity	**7.9**		*CHI3L1*	Extracellular matrix	*78.7*
	**S100A6**	Signal transduction	**19.2**		*CLU*	Complement activity	*78.1*
	**SLC5A12**	Ion transport	**14.9**		*CR2*	Complement activity	*14.4*
	**SPOCK3**	Cell growth survival, maintenance	**7.0**		*CTSL*	Protein metabolism	*38.5*
	**TAGLN2**	Unknown	**7.9**	*	*CYorf15B*	Unknown	*15.9*
	**TBC1D30**	Unknown	**9.7**	*	*DDX3Y*	RNA helicase	*76.3*
	**TNFRSF1B**	Apoptosis inhibitor	**13.7**	*	*DHRS9*	Metabolism	*15.1*
	**TNPO3**	Transcription regulator activity	**11.0**	*	*DMD*	Intracellular signaling	*25.7*
	**TRAF3IP3**	Signal transduction	**5.0**		*DUSP6*	Signal transduction	*34.3*
	**TXN**	Metabolism	**4.1**	*	*EIF1AY*	RNA translation initiator	*104.9*
	**ZNF639**	Transcription regulator activity	**8.2**		*ELL3*	Transcription regulator activity	*11.8*
*	*ALDH2*	Metabolism	*17.2*		*FCGRT*	Signal transduction	*11.9*
	*ALDH5A1*	Metabolism	*4.4*		*FGFR1*	Signal transduction	*5.9*
	*ASAP3*	Cell migration	*7.5*		*FN1*	Extracellular matrix	*17.1*
*	*ASB13*	Intracellular signaling	*11.5*		*GADD45G*	Cell cycle	*8.2*
	*C10orf10*	Activation of transcription	*12.4*	*	*GPD1L*	Unknown, metabolism	*7.3*
*	*CCDC88A*	Cell growth, migration	*8.3*		*GPMS2*	Signal transduction	*9.1*
*	*CD9*	Signal transduction	*29.0*		*GPNMB*	Unknown	*34.8*
	*CDK5R2*	Cell cycle promoter	*5.4*		*GPR137B*	Signal transduction	*10.8*
	*CHAC1*	Apoptosis	*5.3*	*	*IL18*	Cytokine	*87.2*
*	*CYorf15B*	Unknown	*14.1*		*INSR*	Signal transduction	*12.1*
	*DDN*	Unknown	*6.8*	*	*KDM5D*	Minor histocompatibility antigen	*35.4*
*	*DDX3Y*	RNA helicase	*102.9*	*	*LRRC50*	Cell protein assembly	*18.5*
*	*DHRS9*	Metabolism	*14.9*	*	*MEF2B*	Transcription regulator activity	*36.6*
*	*DMD*	Intracellular signaling	*22.3*	*	*MME*	Protein metabolism	*25.5*
*	*EIF1AY*	RNA translation initiator	*195.5*		*MS4A4A*	Unknown	*26.4*
	*ELK3*	Transcription regulator activity	*6.1*		*MYBL1*	Transcription regulator activity	*21.1*
	*FADS3*	Metabolism	*9.1*		*NAIP*	Apoptosis	*10.1*
	*FRZB*	Signal transduction	*115.3*		*NKTR*	Immune response	*4.7*
	*FZD6*	Signal transduction	*7.4*		*PALLD*	Cell growth	*19.4*
	*GP1BB*	Signal transduction	*11.4*		*PAPSS2*	Metabolism	*18.7*
*	*GPD1L*	Unknown, metabolism	*5.7*	*	*PGCP*	Protein metabolism	*13.9*
	*GPR30*	Signal transduction	*27.3*	*	*PLTP*	Cholesterol metabolism	*24.7*
	*IL12RB1*	Cytokine receptor	*3.6*		*PTGDS*	Metabolism	*50.9*
*	*IL18*	Cytokine	*10.0*		*PTK2*	Signal transduction	*17.7*
*	*KDM5D*	Minor histocompatibility antigen	*56.7*	*	*QPCT*	Metabolism	*16.0*
	*LHFPL2*	Intracellular signaling	*3.9*	*	*QPRT*	Metabolism	*13.8*
	*LIMK1*	Intracellular signaling	*5.5*		*RARRES1*	Signal transduction	*60.1*
*	*LRRC50*	Cell protein assembly	*25.9*	*	*RGS13*	Signal transduction	*10.5*
	*LY96*	Signal transduction	*3.8*	*	*RPS4Y*	Protein metabolism	*37.6*
*	*MEF2B*	Transcription regulator activity	*42.4*		*SEPP1*	Metabolism	*479.4*
*	*MME*	Protein metabolism	*42.5*	*	*SLC12A3*	Ion transport	*15.1*
	*NAB1*	Transcription regulator activity	*21.0*	*	*SOCS3*	Signal transduction	*12.0*
	*NAB2*	Transcription regulator activity	*8.3*	*	*SPARCL1*	Signal transduction	*4.4*
	*NET1*	Signal transduction	*4.3*	*	*SPRED2*	Signal transduction	*16.9*
	*NLRP1*	Apoptosis	*5.3*		*STS*	Steroid metabolism	*3.5*
	*PAM*	Protein metabolism	*4.5*	*	*TBL1X*	Signal transduction	*15.5*
*	*PGCP*	Protein metabolism	*5.7*		*TMEM161A*	Unknown	*5.2*
*	*PLTP*	Cholesterol metabolism	*25.8*	*	*TMEM176A*	Unknown	*37.0*
	*PMEPA1*	Signal transduction	*12.0*		*TNFRSF21*	Induction of apoptosis, signal transduction	*21.6*
	*PPIF*	Apoptosis	*8.3*		*TNFSF13*	Induction of apoptosis, signal transduction	*28.3*
	*PPP3CB*	Signal transduction	*3.6*		*TOX*	Transcription regulator activity	*5.8*
*	*QPCT*	Metabolism	*19.9*		*TSPAN13*	Signal transduction	*3.4*
*	*QPRT*	Metabolism	*13.6*		*UBD*	Protein metabolism	*94.5*
*	*RGS13*	Signal transduction	*13.8*	*	*USP9Y*	Protein metabolism	*15.3*
*	*RPS4Y*	Protein metabolism	*36.9*		*VNN2*	Cell migration	*6.1*
*	*SLC12A3*	Ion transport	*9.8*	*	*VPREB3*	Chaperone mu heavy chain	*12.1*
*	*SOCS3*	Signal transduction	*17.4*		*WBP4*	Transcription regulator activity	*5.9*
*	*SPARCL1*	Signal transduction	*6.6*		*XRCC4*	DNA repair	*4.0*
*	*SPRED2*	Signal transduction	*15.7*		*ZBED2*	Unknown	*4.1*
*	*TBL1X*	Signal transduction	*6.4*	*	*ZNF215*	Transcription regulator activity	*11.8*
	*THNSL1*	Metabolism; unknown	*6.3*		*ZNF354A*	Transcription regulator activity	*5.1*
*	*TMEM176A*	Unknown	*6.9*				
	*TRPM4*	Ion transport	*26.8*				
	*TSPAN12*	Signal transduction	*12.5*				
*	*USP9Y*	Protein metabolism	*28.4*				
*	*VPREB3*	Chaperone mu heavy chain	*17.6*				
*	*ZNF215*	Transcription regulator activity	*15.8*				

77 differentially expressed genes in SLE PC compared to tonsil PC and 72 differentially expressed genes in SLE PC compared to tonsil PB, both identified by SAM. The bolded genes represent genes upregulated in SLE PC compared to tonsil Ig secreting cells. The genes in italics represent downregulated genes compared with tonsil Ig secreting cells. The asterisk denotes genes common to both comparisons. Gene function adapted from NCBI Gene and HPRD (Human Protein Reference Database).

### Comparison of SLE PC and Bone Marrow PC

**Figure 5:**
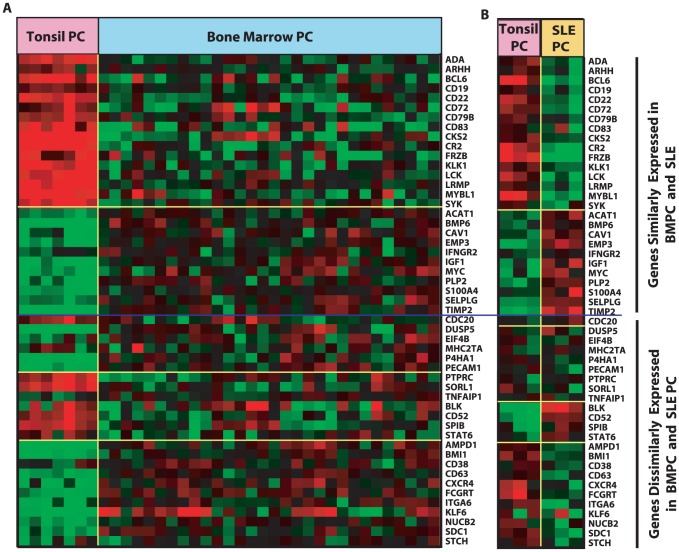
Comparative expression of bone marrow PC discriminating genes in SLE PC and tonsil PC. 51 genes previously reported to discriminate bone marrow PC from tonsil PC (**A**) were compared to gene expression profiles of SLE PC and tonsil PC from the current data set (**B**). The blue line in the figure divides the 51 genes into those that are similarly (top) and dissimilarly (bottom) expressed by SLE PC and bone marrow PC. Twenty-seven genes demonstrate a similar pattern of expression in SLE PC and bone marrow PC and 24 genes exhibit a dissimilar pattern of expression in bone marrow and SLE PC.

We were next interested in determining whether SLE PC resembled a more mature PC, such as the bone marrow PC, because of several features of SLE PC, including the degree of downregulation of expression of MHC Class II, cell surface markers and genes involved in limiting apoptosis and arrested cell cycle. To carry out this comparison, we analyzed the previously published normal bone marrow PC differential gene expression program [22] and compared it with gene expression seen in SLE PC and tonsil PC. We focused on 51 of the most discriminating bone marrow PC genes previously cited [21], [22] for which we had equivalent probe sets available. Of the 51 genes analyzed, 27 exhibited similar expression in SLE PC and bone marrow PC but differed from the tonsil comparators as shown in **Figure 5** suggesting the expression of these genes as similar in bone marrow and SLE PC. Expression of the remaining 24 genes in SLE PC and tonsil PC was variable with 8 genes that were expressed equivocally, 4 genes with greater expression in SLE PC than tonsil PC and 11 genes with less expression in SLE PC as compared to tonsil PC, and thus different from the published comparison with tonsil PC and bone marrow PC. The genes expressed with a high degree of concordance between SLE PC and bone marrow PC include *ADA*, *BCL6*, *CD79*, *CR2*, *CD19*, *CD22*, *LCK*, *SYK*, *CAV1*, *EMP*, *MYC* and *S100A4*. Genes whose expression was discordant between SLE PC and bone marrow PC include *CXCR4*, *ITGA6*, *SDC1*, *CD63*, *FCγRT*, *STCH*, *CD38*, *TNFAIP1*, *MHC2TA* and *PECAM1*. The genes with the most striking discordance between SLE PC and bone marrow are *CXCR4*, *SDC1*, *CD63*, *ITGA6* and *FCγRT*. SLE PC had very low expression of these genes. Taken together these results suggest that SLE PC have gene expression profiles with similarities to both tonsil PC and bone marrow PC, and may, therefore, be at a stage of differentiation between recently generated tonsil PC and more mature bone marrow PC.

### SLE Peripheral Blood B cells have an upregulated type 1 IFN inducible gene signature

**Figure 6:**
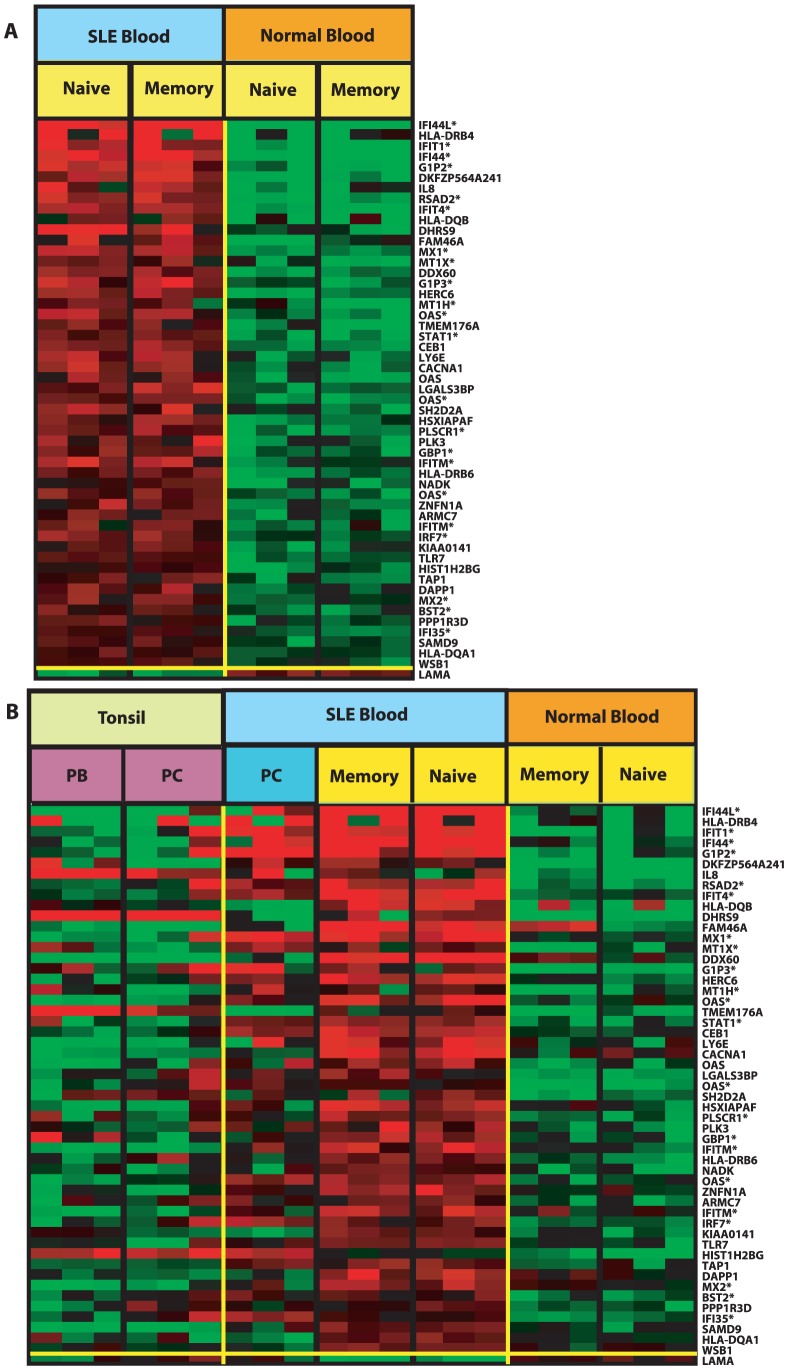
The IFN signature in SLE B cell subpopulations. **A**. A total of 54 genes identified using SAM are differentially expressed in SLE naïve and memory B cells compared with normal comparators. Fifty-three of the 54 genes are upregulated and 23 (denoted by an asterisk) comprise the IFN inducible gene signature. **B.** The expression of the genes shown in part A was assessed for corresponding expression in IgSC populations.

Peripheral blood mononuclear cells from SLE patients have been shown to express a type 1 interferon (IFN) inducible gene signature [33], [34]. We next interrogated circulating SLE B cell lineage subsets to determine whether they also expressed the interferon signature. When SLE naïve and memory B cells were assessed, a total of 53 genes were found to be differentially expressed compared with normal naïve and memory B cells, with 52 differentially upregulated (using SAM as previously stated) as shown in **Figure 6a**. The differential gene expression recapitulates an upregulation of type 1 IFN inducible genes with 23 of the 53 genes known to be IFN inducible genes. In addition to IFN inducible genes, a limited number of MHC class II were also upregulated as well as genes involved in antigen presenting capacity (*TAP1*, *DAPP1*) and genes involved in B cell survival and proliferation, such as *BST2*. In addition, *LY6E*, a biomarker of disease activity in SLE was upregulated in SLE B cells [35]. We next sought to determine if these 53 differentially expressed genes, including the interferon signature, were expressed at similar level in SLE PC and tonsil Ig secretors. As can be seen in **Figure 6b**, several of the IFN inducible genes were upregulated in SLE PC, but not tonsil PC or PB. These include *IFI44L*, *IFIT1*, *G1P2*, *IFIT4*, *RSAD2*, *MX1*, *MT1X*, *STAT1*, *OAS*, *IRF7*, *IFI35* and *BST2*. *HIST1H2BG* appeared to have similar expression in all IgSC and is differentially upregulated in tonsil PC compared to tonsil non Ig secreting B cells (**Figure 1** and **Table S1**) and SLE PC compared to the remaining SLE B cells (**Figure 2** and **Table S2**). *DHRS9* and *TMEM176A* are downregulated in the SLE PC compartment and expressed differentially compared to tonsil PC and tonsil PB (**Table 2**). Thus, *DHRS9* and *TMEM176A* appear to be downregulated specifically in SLE PC, as they are expressed at greater levels in both tonsil IgSC populations. *DHRS9* is upregulated SLE naïve and memory B cells and in tonsil PC compared with other tonsil B cells (**Figure 1** and **Table S1**). The genes *DDX60*, *CACNA1A*, *KIAA0141* and *HLADQB1* are differentially upregulated in the SLE naive and memory compartments compared to normal naive and memory B cells and are differentially downregulated in SLE PC compared to SLE naïve and memory B cells (**Figure 2**). It should be noted that no interferon inducible genes were identified as differentially expressed between tonsil PC versus PB and either tonsil PC or tonsil PB versus SLE PC.G1P3 alone was found to be differentially upregulated in tonsil PC compared with tonsil non-IgSC, demonstrating that normal IgSC do not possess an IFN inducible signature. Furthermore, when SLE PC were compared with tonsil non-IgSC, several IFN inducible genes were highly and significantly upregulated (greater than 2 fold, p<0.001). These genes are *RSAD2*, *G1P2*, *IFI35*, *IFIT1*, *IRF7*, *MT1X*, *MX1*, *OAS1*, *OAS2* and *OASL* (data not shown). Therefore the IFN inducible gene signature is limited to the SLE disease state.

## Discussion

SLE is a complex and heterogeneous disease with known alterations in the phenotype of circulating T and B cells, granulocytes, monocytes and dendritic cells [7], [33], [34], [36], [37], [38], [39], [40]. The peripheral B cell compartment in SLE is particularly abnormal and known to have increased circulating PC, whose presence correlates with pathogenic autoantibody production. Recently, therapeutic interventions aimed at depleting the B cell population have been attempted in SLE [41], [42] with mixed overall therapeutic benefit. Despite the observation of increased circulating PC in SLE, comprehensive molecular profiling has not yet been reported in this B cell population in SLE. The approach that we employed involved molecular profiling of isolated B cell subsets from subjects with active SLE, followed by comparison of the molecular profiles with PC at specific stages of differentiation. This permitted a specific molecular identification of the differentiation stage of circulating SLE PC and also provided some new insights into the possible mechanisms for their persistence in the blood.

Initially, we analyzed tonsil PC and PB in order to establish a common molecular gene expression profile of these IgSC populations. This signature demonstrated the differential expression of many genes involved in the biologic function of IgSC and was similar to that reported previously in either gene expression studies or studies of the essential function of specific gene products in IgSC biology [15], [16], [22], [24], [25], [26], [43], [44], [45]. Importantly, differences between the gene expression profiles of PB and PC were also noted. The differential expression of genes promoting apoptosis and certain cell surface markers as well as those affording protection from apoptosis by limiting ER stress are all consistent with the conclusion that PC and PB are indeed separate populations that differ with regard to the stage of differentiation. By using gene expression profiles of PB and PC as a reference, we were able to characterize circulating SLE PC as more similar to the more mature tonsil PC population. In this regard, SLE PC had the typical gene expression profile of IgSC shared with both reference populations, but also more of the unique features of post-switch tonsil PC than of pre-switch tonsil PB. The genes upregulated and differentially expressed by both SLE PC and tonsil PC include *ATF6*, *ESR1*, *IRF4*, *XBP1*, *SSR3* and chaperones *(DNAJB4*, -*5*, -*9* and *DNAJC1*), whereas the downregulated genes included *SPIB*, *CD22*, *BLK* and *FYN* for example, indicating that both share a more mature molecular phenotype than tonsil PB.

It has been reported that the long-lived potential of a PC is closely related to the expression of genes that control ER stress (*XBP1*, UPR and chaperones) [12], [13]. In addition, the high expression of *PRDM1* (encoding Blimp1) is associated with survival of PC. In contrast, the inactivation of *PRDM1* has been shown to result in the disappearance of PC from the bone marrow [12]. *PRDM1* increases the expression *XBP1* and the inhibition of *XBP1* can lead to ER stress induced apoptosis [44], [45]. The tonsil PC, PB and SLE PC have very similar expression of these genes indicating that may have a comparably important role in maintaining all IgSC populations.

The gene signature of SLE PC was not completely consonant with that of tonsil PC, but rather showed some features of the previously published molecular profile of the mature bone marrow PC. Bone marrow PC exhibit a gene signature profile characterized by low expression of cell surface markers, elevated expression of genes regulating an anti-apoptotic program and promotion of cell cycle arrest [46]. The graded stages of increasing maturation in mature human PC are generally accepted as location specific with tonsil, blood and then bone marrow as the most mature PC. Comparison with the PC gene expression profiles within these compartments provided a useful approach to stage the maturation status of SLE PC. Of the genes examined whose expression has been reported to be characteristic of bone marrow PC, approximately half were similarly expressed in SLE PC and not tonsil PC. The genes expressed similarly in bone marrow and SLE PC but not in tonsil PC include *ADA*, *BCL6*, *MYC*, *LCK*, *SYK*, *ACAT1*, *S100A4*, *CAV1* and surface markers *CD19*,-*22*, -*72*, -*79* and *CD83*. Notably, in SLE PC, expression of some genes did not match that expected from the bone marrow PC profile, including *CXCR4*, *ITGA6* and *PECAM1*, which may reflect abnormal migration of SLE PC to tissue niches. From the current studies we cannot determine whether these differences are specific to SLE or rather reflect differences in the stage of maturation of blood versus tonsil and bone marrow PC.

A few features of SLE PC are noteworthy. The first relates to the expression of MHC class II genes, which is very low in SLE PC. Diminished expression of MHC II has previously been described as a feature of mature PC [26], [46], [47]. Part of this may relate to the markedly increased expression of *PRDM1* that encodes BLIMP-1. This transcriptional repressor exerts many actions on PC, one of which is to negatively regulate major histocompatibility class II transactivator (*MHC2TA*) which regulates MHC class II surface expression [26]. *MHC2TA*, along with most MHC II genes were markedly decreased in SLE PC, and to a greater degree than in either tonsil PC or PB. This suggests a possible mechanism for the decrease in MHC II genes and is also consistent with the mature status of SLE PC.

The finding of markedly decreased MHC II genes in SLE PC is somewhat different than previously reported results examining the phenotype of SLE PC by flow cytometry. This report noted that the circulating PC found in lupus patients with active disease were HLA-DR expressing PB and that the frequency of these cells might correlate with disease activity and autoantibody titers [2]. It should be noted that although this report focused on PB, both PB and MHC-II^dim^ PC were found to be increased in active SLE. Moreover, increased numbers of circulating PC have been reported in SLE subjects with chronic smoldering disease as well as in those with acute flares of disease activity [48]. There are a few possible explanations for these discrepancies. First, the previous study examined cell surface expression of MHC II whereas the current study examined mRNA levels. Since SLE PC are likely to be in a dynamic state of differentiation, it is possible that mRNA levels had already declined but protein expression persisted. Secondly, the previous study may have examined subjects with a greater degree of disease activity. It is, therefore, possible that in the chronic state increased number of mature PC are found in the circulation whereas during acute exacerbations, additional MHC class II positive PB are generated which contribute to the circulating IgSC pool.

**Figure 7:**
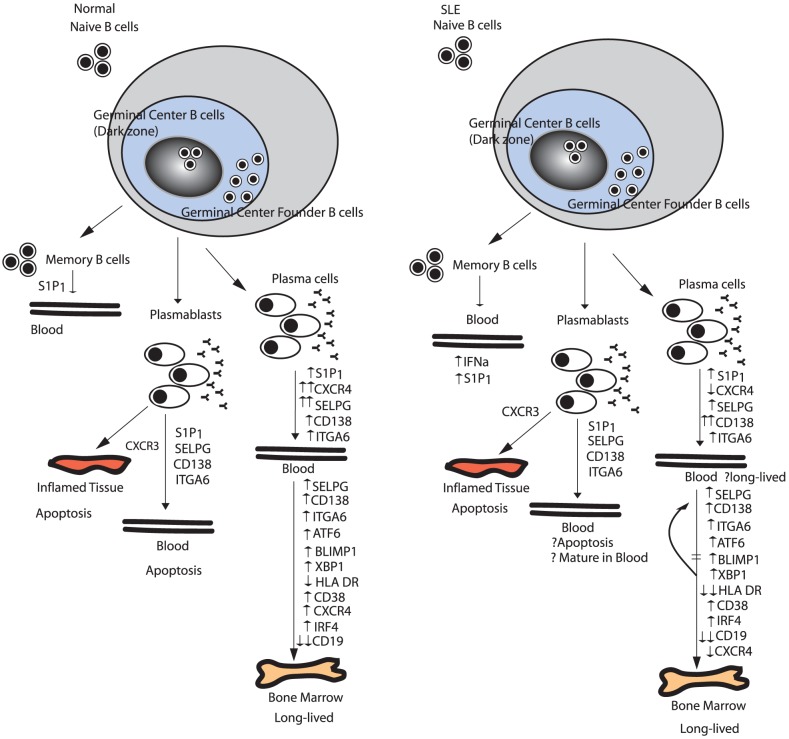
Proposed plasma cell trafficking in SLE based on gene expression profiling. Normal B cell traffic into germinal center reactions appears unchanged in SLE from normal. Plasma cells in SLE differ from tonsil plasma cells in expression of cell surface markers that may affect plasma cell homing to bone marrow. Plasma cells continue to mature in the blood and have a gene program upregulated similar to bone marrow plasma cells with long-lived potential. Downregulated gene program include continued and more pronounced decrease in expression of CXCR4, MHC class II and cell surface markers. Endogenous factors such as IFN*α* in SLE may contribute to these changes.

Most available data are consistent with the conclusion that circulating PC in SLE arise in T cell dependent GC reactions in secondary lymphoid organs. Consistent with this is the highly mutated nature of their Ig heavy chain genes and the finding that they rapidly decrease following administration of a blocking monoclonal antibody to CD154 that limits T cell-B cell collaboration and germinal center formation [6]. One of the unresolved questions, however, remains why they persist in the circulation. The current analysis may have provided some clues. It is known that normal circulating PC have higher gene and cell surface expression of CXCR4 [22], [49]. In contrast, the current data demonstrate that circulating SLE PC has very low expression of CXCR4 mRNA. Interaction between CXCR4 and its ligand CXCL12 is known to be important not only for the homing of PC to bone marrow survival niches, but also for survival of bone marrow PC [11], [50]. It is, therefore, possible that SLE PC are less able to migrate into survival niches because of their decreased expression of CXCR4, thereby contributing to their persistence in the circulation. Figure 7 outlines key regulators in plasma cell trafficking and the proposed differences in normal and SLE disease state and the effects on plasma cell homing and life span.

S1P_1_ expression also plays a role in mobilizing lymphocytes from secondary lymphoid organs [51]. Moreover, the relative density of CD69 contributes to the regulation of S1P_1_ expression by forming a complex with and negatively regulating S1P_1_ to promote lymphocyte retention in lymphoid organs. It is notable that expression of both CD69 and S1P_1_ was elevated in SLE PC, possibly also contributing to abnormalities in PC homing behavior.

The IFN signature, comprised of IFN inducible genes, has previously been reported to be upregulated in SLE and other autoimmune and inflammatory diseases. We found that the IFN signature was upregulated on all SLE B cell populations, including PC. The genes comprising this signature are similar to the previously reported IFN inducible genes [33], [34]. The IFN signature found in SLE PC does not necessarily imply a role for type 1 IFN in PC differentiation since the signature was found on all SLE B cell populations examined and even more prominently on non-IgSC and was not found on tonsil PC. Whether type 1 IFN contributes directly or indirectly to the increased activity of B cells in SLE is a matter of current debate, but certainly the signature can be easily detected in B lineage cells.

In summary, SLE PC possess a gene expression profile most consistent with that of mature PC, having features of both tonsil and bone marrow PC. Although the IFN signature is also expressed by SLE PC, it is not more prominent than expressed by other B lineage cells in SLE. Together, the data have defined the molecular nature of the circulating PC in SLE and provided some clues for the reasons for the persistence of these cells in the circulation of subjects with SLE.

## Supporting Information

Table S1 **List of genes in the order they appear in** **Figure 1** **Heatmap.** The column heading Log 2 difference is the difference in gene expression between Tonsil plasma cells (Tonsil PC) and the remaining tonsil non-Ig secreting B cells (Naïve; GCF- germinal center founder/activated naïve; GC- germinal center; Memory). Fold change represents the fold change for the log 2 difference calculated. Yellow highlighted genes represent the probe sets and corresponding genes that appear more than once as differentially expressed genes. Black font represents genes upregulated in Tonsil PC compared to the remaining tonsil non-Ig secreting B cells and red font are genes that are differentially downregulated in Tonsil PC compared to the remaining tonsil non-Ig secreting B cells.
(XLSX)

Table S2 **List of genes in the order they appear in** **Figure 2** **Heatmap.** The column heading Log 2 difference is the difference in gene expression between SLE plasma cells and SLE naive and memory B cells. Fold change represents the fold change for the log 2 difference calculated. Yellow highlighted genes represent the probe sets and corresponding genes that appear more than once as differentially expressed genes. Black font represents genes upregulated in SLE plasma cells compared to SLE naive and memory B cells and red font are genes that are differentially downregulated in SLE plasma cells compared to SLE naive and memory B cells.
(XLSX)
